# Osteopontin Upregulates the Expression of Glucose Transporters in Osteosarcoma Cells

**DOI:** 10.1371/journal.pone.0109550

**Published:** 2014-10-13

**Authors:** I-Shan Hsieh, Rong-Sen Yang, Wen-Mei Fu

**Affiliations:** 1 Department of Pharmacology, College of Medicine, National Taiwan University, Taipei, Taiwan; 2 Department of Orthopedic Surgery, National Taiwan University Hospital, Taipei, Taiwan; National Center for Scientific Research Demokritos, Greece

## Abstract

Osteosarcoma is the most common primary malignancy of bone. Even after the traditional standard surgical therapy, metastasis still occurs in a high percentage of patients. Glucose is an important source of metabolic energy for tumor proliferation and survival. Tumors usually overexpress glucose transporters, especially hypoxia-responsive glucose transporter 1 and glucose transporter 3. Osteopontin, hypoxia-responsive glucose transporter 1, and glucose transporter 3 are overexpressed in many types of tumors and have been linked to tumorigenesis and metastasis. In this study, we investigated the regulation of glucose transporters by osteopontin in osteosarcoma. We observed that both glucose transporters and osteopontin were upregulated in hypoxic human osteosarcoma cells. Endogenously released osteopontin regulated the expression of glucose transporter 1 and glucose transporter 3 in osteosarcoma and enhanced glucose uptake into cells via the αvβ3 integrin. Knockdown of osteopontin induced cell death in 20% of osteosarcoma cells. Phloretin, a glucose transporter inhibitor, also caused cell death by treatment alone. The phloretin-induced cell death was significantly enhanced in osteopontin knockdown osteosarcoma cells. Combination of a low dose of phloretin and chemotherapeutic drugs, such as daunomycin, 5-Fu, etoposide, and methotrexate, exhibited synergistic cytotoxic effects in three osteosarcoma cell lines. Inhibition of glucose transporters markedly potentiated the apoptotic sensitivity of chemotherapeutic drugs in osteosarcoma. These results indicate that the combination of a low dose of a glucose transporter inhibitor with cytotoxic drugs may be beneficial for treating osteosarcoma patients.

## Introduction

Osteosarcoma is the most common type of bone cancer in teenagers and is highly metastatic [1], [2]. Surgery and chemotherapy are the standard treatment options for high-grade osteosarcoma. However, approximately 20% of patients have lung metastases at initial diagnosis and 40% patients experience metastasis at a later stage. The 5-year survival rate for osteosarcoma patients with metastases is 20%, compared with 65% for patients with localized disease [3].

Glucose is a source of metabolic energy that maintains tumor cells’ ability to proliferate and survive. Glucose transporters (GLUTs) move glucose into the cytosol to fuel aerobic glycolysis, also known as the Warburg effect [4]–[6]. GLUT1 and GLUT3 are class I glucose transporters that possess a high affinity for glucose and are hypoxia-responsive. Hypoxia is an important factor during tumor progression. Under hypoxic conditions, HIF-1 (hypoxia-inducible factor 1) regulates the expression of numerous genes, such as VEGF (vascular endothelial growth factor), iNOS, EPO, LDHA (lactate dehydrogenase A), PDK1 (pyruvate dehydrogenase kinase 1), GLUT1, and GLUT3 [7]. Expression of GLUT1 and GLUT3 is regulated by developmental stage and metabolic state. Upregulation of GLUT1 and GLUT3 are reported to be associated with poor prognosis in breast cancer [8]. Overexpression of GLUT1 also corresponds with poor survival in non-small cell lung cancer [9] and tumor aggressiveness in transitional cell carcinoma of the bladder [10]. Many cancers overexpress GLUTs because of the energy requirement associated with uncontrolled proliferation and metastasis [11]; however, few studies examine the relationship between osteosarcoma progression and GLUTs.

Osteopontin (OPN) is a noncollagenous bone matrix protein that earned its name from its discovery in osteoblasts [12], [13]. OPN interacts with cells through many different integrins, including αvβ1, αvβ3, and αvβ5, via the GRGDS. OPN also binds to the CD44 receptor on the cell membrane to regulate cytokine production, cell trafficking, cell proliferation, migration, and cell survival [14], [15]. OPN expression is associated with the progression of several cancers, including breast, ovarian, prostate, renal, oral, colorectal, pancreatic, liver, lung, skin, and thyroid cancers, glioblastoma, and sarcomas. The interaction of OPN with various receptors, including several integrins and CD44, induces the activation of signal transduction pathways leading to cell migration and invasion. The level of OPN is also related to tumor stage and is a biomarker for cancer progression and prognosis in many cancers. The upregulation of OPN levels concomitant with cancer type-specific markers aids in early detection of many malignancies [16]–[18]. VEGF, THBS3 (thrombospondin 3), osteocalcin, osteonectin, VS38c, and S100 are specific markers for osteosarcoma [19]. Higher levels of these markers are detected in the peripheral blood of osteosarcoma patients [20]. Overexpression of OPN also occurs in many osteosarcoma samples [21]. Although osteopontin has multiple physiological functions, including the attachment of osteogenic cells to the bone matrix, control of mineralization, coupling of bone formation, and resorption [22], however, the role of OPN in osteosarcoma is still not clear. In this study, we found that GLUTs and OPN increased during hypoxic conditions in osteosarcoma. OPN upregulated GLUT1 and GLUT3 expression via αvβ3 integrin and the AKT, JNK, and p38 pathways in osteosarcoma cells. Knockdown of OPN increased cell death in osteosarcoma cell lines. Chemotherapeutic drugs in combination with a low dose of glucose transporter inhibitor exerted synergistic cytotoxic action. Taken together, these data suggest a new therapeutic strategy for osteosarcoma.

## Materials and Methods

### Cell culture

The human osteosarcoma cell lines MG63, U-2OS, and 143B were purchased from the American Type Culture Collection (Rockville, MD). MG63 cells were cultured with DMEM (Gibco; Grand Island, NY), U-2OS cells were cultured with RPMI 1640, and 143B cells were cultured with MEM. All cell cultures were supplemented with 10% fetal bovine serum (FBS; Hyclone, Logan, UT) and maintained at 37°C in a humidified atmosphere with 5% CO_2_.

### RNA interference

An OPN-shRNA (short-hairpin RNA) conjugated to the pLKO.1 vector containing a puromycin-resistant region was provided by the National RNAi Core Facility at the Institute of Molecular Biology/Genomic Research Center in Taipei in Taiwan. The sequences as shown below:

OPN-shRNA #1:

CCGG**CTTCAGGGTTATGTCTATGTT**CTCGAGAACATAGACATAACCCTGAAGTTTTT

OPN-shRNA #2:

CCGG**CCACAAGCAGTCCAGATTATA**CTCGAGTATAATCTGGACTGCTTGTGGTTTTT

Control-shRNA:

CCGG**TCACAGAATCGTCGTATGCAG**CTCGAGCTGCATACGACGATTCTGTGATTTTTG

shRNA plasmids and TurboFect Transfection Reagent (#R0531; Thermo Scientific) were premixed with Opti-MEM I (Gibco, Grand Island, NY) separately for 5 min, mixed with each other for 25 min, and then applied to MG63 and U-2OS cell cultures. The control shRNA (empty vector; ev) was used as a negative control. For transient transfection, cells were transfected with two different OPN-shRNA plasmids for 24 h.

### Western blot

After washing with cold phosphate-buffered saline (PBS), cells were lysed with 50 µl radioimmunoprecipitation assay buffer [RIPA; 50 mM HEPES, 150 mM NaCl, 4 mM EDTA, 10 mM Na_4_P_2_O_7_, 100 mM NaF, 2 mM Na_3_VO_4_, 1% Triton X-100, 0.25% sodium deoxycholate, 50 mM 4-(2-aminoethyl)-benzene sulfonylfluoride, 50 µg ml^−1^ leupeptin, 20 µg ml^−1^ aprotinin, pH 7.4] on ice for 30 min. Following centrifugation of lysates at 14,500 r.p.m. for 1 h, the supernatant was isolated and used for western blotting. Protein concentration was measured using a BCA assay kit (Pierce, Rockford, IL) with bovine serum albumin as a standard. Equal concentrations of protein were separated on 8% sodium dodecyl sulfate-polyacrylamide (SDS) gels and transferred to nitrocellulose membranes (Millipore, Bedford, MA, USA). The membranes were incubated for 1 h with 5% dry skim milk in PBS buffer to block nonspecific binding and then incubated overnight at 4°C with the following primary antibodies: rabbit anti-GLUT-1, 2, 3, and 4 (1∶1,000; Millipore, Billerica, MA), anti-OPN (1∶1,000; Abcam Inc., Cambridge, MA), and mouse anti-β-actin (1∶10,000; Santa Cruz Biotechnology, Dallas, TX). After washing with phosphate buffered saline Tween (PBST), the membranes were then incubated with mouse anti-rabbit or goat anti-mouse peroxidase-conjugated secondary antibody (1∶1,000; Santa Cruz Biotechnology, Dallas, TX) for 1 h. The blots were visualized by enhanced chemiluminescence (ECL; Millipore, Billerica, MA) using a UVP imaging system (UVP, Upland, CA). For normalization purposes, each blot was also probed with mouse anti-β-actin (1∶10,000; Santa Cruz Biotechnology, Dallas, TX).

### Quantitative Real-Time PCR

Total RNA was extracted using a TRIzol kit (MDBio, Inc., Taipei, Taiwan). 2 µg RNA was used for reverse transcription that was performed with a commercial kit (Invitrogen, Carlsbad, CA). Quantitative real-time PCR was performed using a TaqMan/SYBR Master Mix (Thermo Scientific) and analyzed with a model StepOne plus System (Applied Biosystems; Foster City, CA). After pre-incubation at 50°C for 2 min and 95°C for 10 min, the PCR was performed at 40 cycles of 95°C for 15 sec and 60°C for 1 min. The threshold was set above the non-template control background and within the linear phase of target gene amplification to calculate the cycle number at which the transcript was detected (denoted as CT). The cDNA was amplified with gene specific primers as shown below:

GLUT1:

Forward: CCAGC TGCCA TTGCC GTT

Reverse: GACGT AGGGA CCACA CAGTT GC

GLUT2:

Forward: CACAC AAGAC CTGGA ATTGA CA

Reverse: CGGTC ATCCA GTGGA ACAC

GLUT3:

Forward: CAATG CTCCT GAGAA GATCA TAA

Reverse: AAAGC GGTTG ACGAA GAGT

GLUT4:

Forward: CTGGG CCTCA CAGTG CTAC

Reverse: GTCAG GCGCT TCAGA CTCTT

GLUT6:

Forward: GCCCG GACTA CGACA CCT

Reverse: AGCTG AAATT GCCGA GCAC

GLUT8:

Forward: TCATG GCCTT TCTCG TGAC

Reverse: TCCTT TAGTT TCAGG GACAC AG

GLUT10:

Forward: CTGTG GAGAT ACGAG GAAGA

Reverse: TCAGT CCGTA GAGCA GGA

GLUT12:

Forward: GGTAC CTGTT GAAAA CACCG

Reverse: GCAGT GACAG ATGAC AGGAA

OPN:

Forward: CTGTG CCATA CCAGT TAA

Reverse: GATGT CAGGT CTGCG AAA

GAPDH:

Forward: CAGAA CATCA TCCCT GCCTC T

Reverse: GCTTG ACAAA GTGGT CGTTG AG

TagMan probes (Applied Biosystems; Foster City, CA)

SPP1 (OPN): Hs 00167093_ml

SLC2A1 (GLUT1): Hs 00892681_ml

SLC2A3 (GLUT3): Hs 00359840_ml

SLC2A4 (GLUT4): Hs 00168966_ml

GAPDH: Hs 99999905_ml

### Cell viability assay

Cell viability was assessed by MTT [3-(4,5-dimethyl thiazol-2-yl)-2,5-diphenyl tetrazolium bromide] (Sigma-Aldrich, St. Louis, MO) assay. The culture medium was aspirated 24 h after drug treatment and MTT (0.5 mg/ml) was added to each well. The MTT was removed after 30 min and the cells were lysed using 100 µl dimethylsulfoxide (DMSO). The absorbance was measured at 550 nm and 630 nm using a microplate reader (Bio-Tek, Winooski, VT).

### Glucose uptake assay

Glass coverslips were coated with poly-d-lysine for 1 h at room temperature and then rinsed with sterile d.d. H_2_O (3 times/5 min). Cells were seeded onto coverslips for 24 h. They were then treated with OPN (100 ng/ml) for 24 h followed by 2-deoxy-d-glucose (2-NBDG) for 30 min at 37°C. After uptake of 2-NBDG, the cells were put on ice and fixed with 4% paraformaldehyde in PBS for 15 min at 4°C. Images were obtained from a fluorescent microscope using an excitation wavelength of 485 nm and an emission wavelength of 535 nm (model SP5 TCS; Leica, Heidelberg, Germany).

### Flow cytometry

The effect of OPN on the cellular uptake of 2-NBDG was also measured by flow cytometry. Briefly, 5×10^5^ cells were incubated in 6-well plates for 24 h. The cells were treated with OPN (100 ng/ml) at 37°C for 24 h. The cells were then detached by trypsin and 2-NBDG was added and incubated at 37°C for another 0.5 h. The cells were collected and washed twice with ice-cold PBS buffer. Finally, the cells were resuspended in cold PBS buffer for flow cytometric analysis. The relative values of 2-NBDG staining intensity were obtained by dividing the fluorescence intensity of each measurement by that of control cells.

### Statistics

Values are expressed as the mean ± S.E.M from at least three experiments. Results were analyzed with one-way analysis of variance (ANOVA), followed by Student’s *t*-test. Significance was defined as *p*<0.05.

## Results

### Hypoxia increases osteopontin expression in human osteosarcoma cells

**Figure 1:**
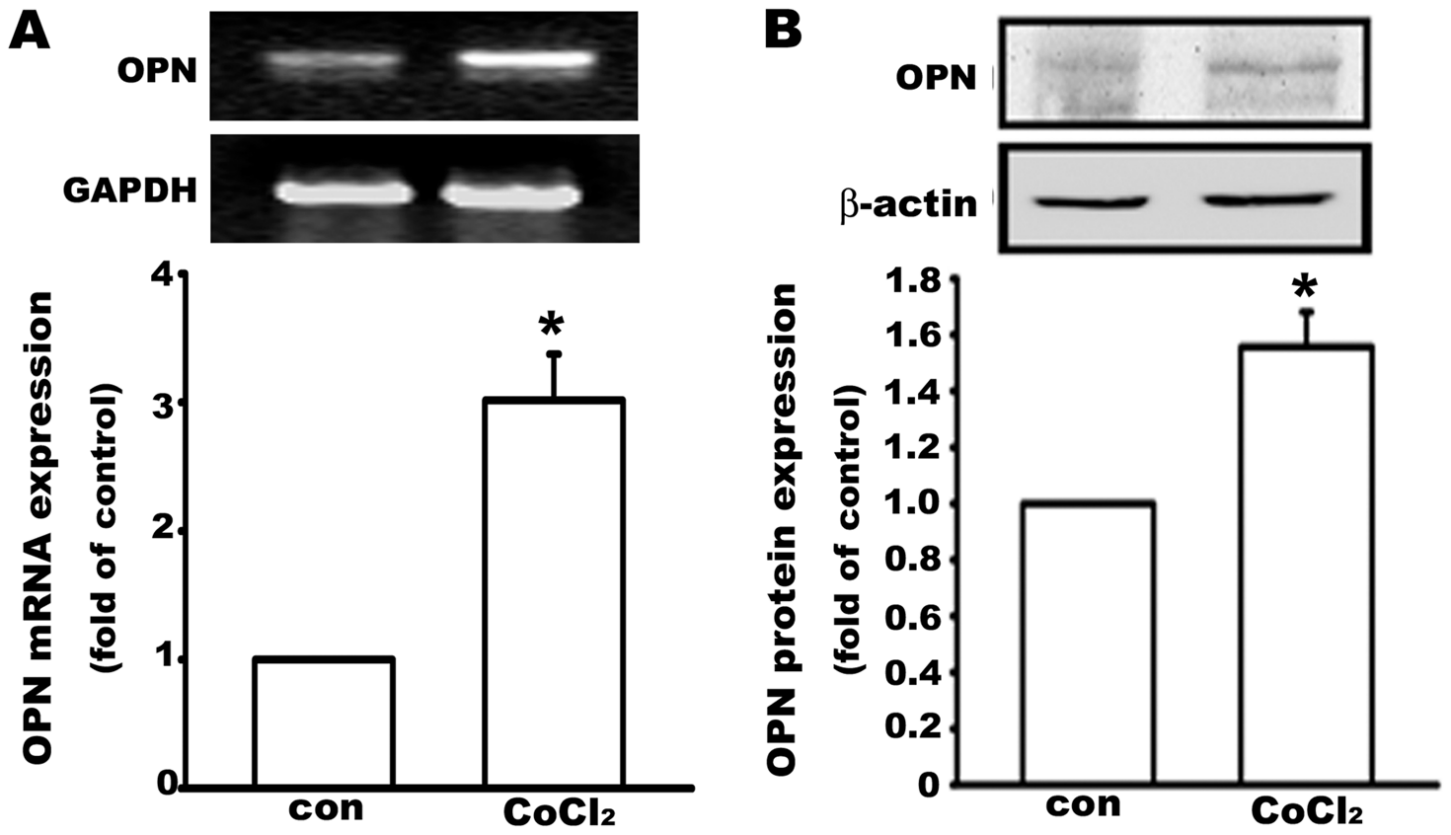
Hypoxia increases osteopontin expression in human osteosarcoma cells. MG63 osteosarcoma cells were treated with the chemical hypoxic agent CoCl_2_ (100 µM). Osteopontin (OPN) mRNA (6 h) (A) and protein (24 h) (B) levels were increased by CoCl_2_ treatment. Data are presented as the mean ± S.E.M. (n = 3), *p≤0.05, as compared with the control (con).

Hypoxia is a major regulator of tumor development and aggressiveness [23]. Osteopontin levels are also known to be upregulated in a variety of cancers. A dose of 100 µM cobalt chloride (CoCl_2_), a hypoxia-mimetic agent that induces HIF1α expression, stabilization, and activation, was used to mimic the hypoxia seen during tumor development. We observed that osteopontin mRNA (6 h, Figure 1A) and protein (24 h, Figure 1B) levels were markedly increased in MG63 human osteosarcoma cells after treatment with CoCl_2_, indicating that osteopontin may play a role in osteosarcoma progression.

### Hypoxia increases the expression of glucose transporters in human osteosarcoma cells

**Figure 2:**
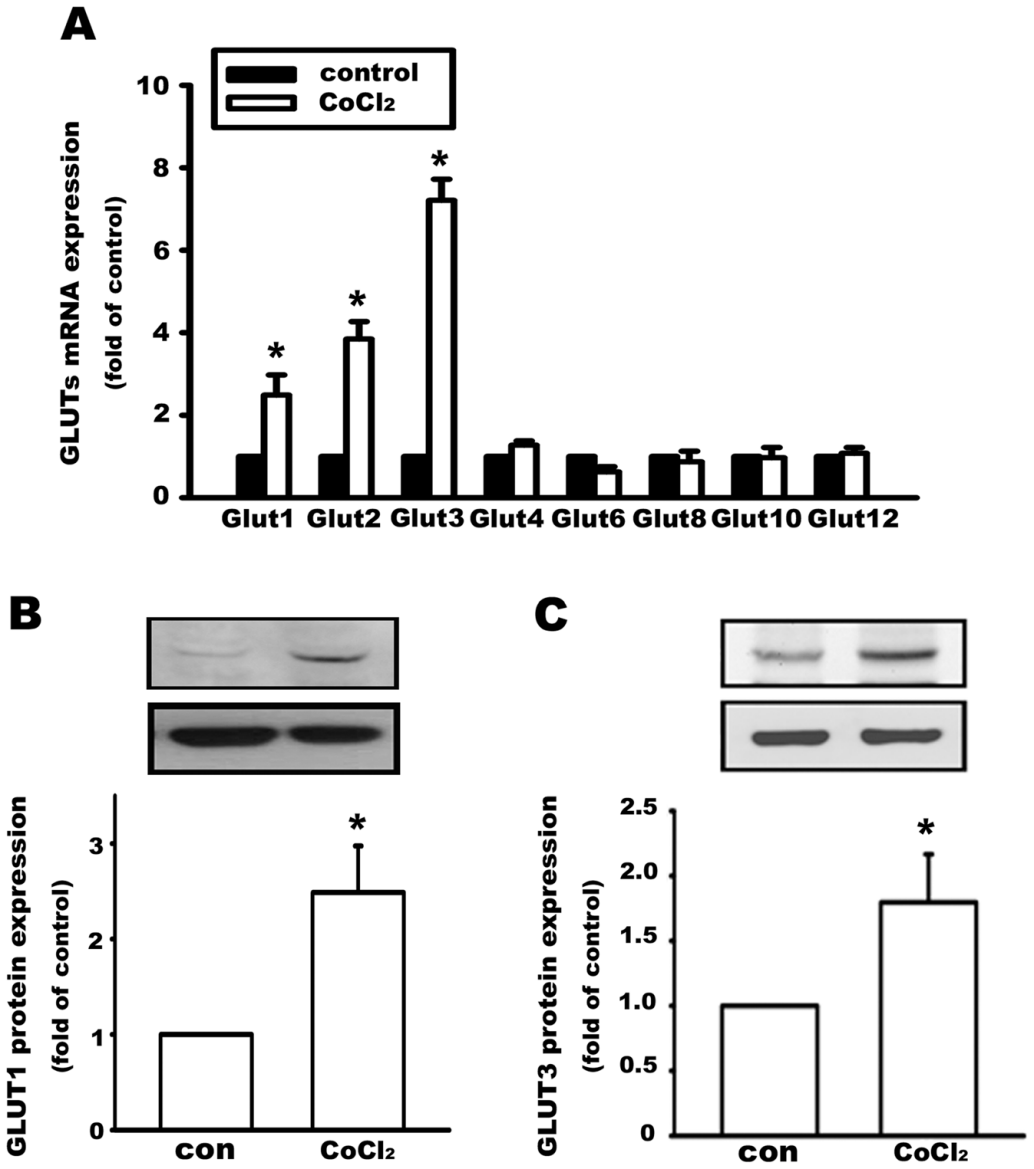
Hypoxia increases the expression of glucose transporters in human osteosarcoma cells. (A) The mRNA levels of glucose transporter (GLUT) 1, 2, 3, 4, 6, 8, 10, and 12 were evaluated using quantitative PCR. After treatment with CoCl_2_ (100 µM, 6 h), GLUT 1, 2, and 3 mRNA levels were increased. CoCl_2_ (100 µM, 24 h) also increased GLUT1 (B) and GLUT3 (C) protein levels in MG63 cells. Data are presented as the mean ± S.E.M. (n = 3), *p≤0.05, compared with the control group (con).

Glucose transporters are another common regulator of tumor growth [5], [24]. Tumor cells turn on the hypoxia-inducible transcription factor oxygen-sensing system and regulate the downstream genes, such as VEGF, iNOS, EPO, GLUT1, and GLUT3, to adapt to hypoxia and increase tissue oxygenation [7], [25]–[27]. In MG63 human osteosarcoma cells, the mRNA levels of GLUT1, GLUT2, and GLUT3, but not GLUT 4, 6, 8, 10, or 12, were increased after a 6-h treatment of CoCl_2_ (100 µM) (Figure 2A). Because GLUT1 and GLUT3 possess a high affinity for glucose, we then measured the protein expression of GLUT1 and GLUT3. We observed that protein levels of GLUT1 and GLUT3 were upregulated after treatment of CoCl_2_ (100 µM, 24 h) (Figures 2B and 2C).

### Osteopontin increases GLUT1 and GLUT3 expression in osteosarcoma cells

**Figure 3:**
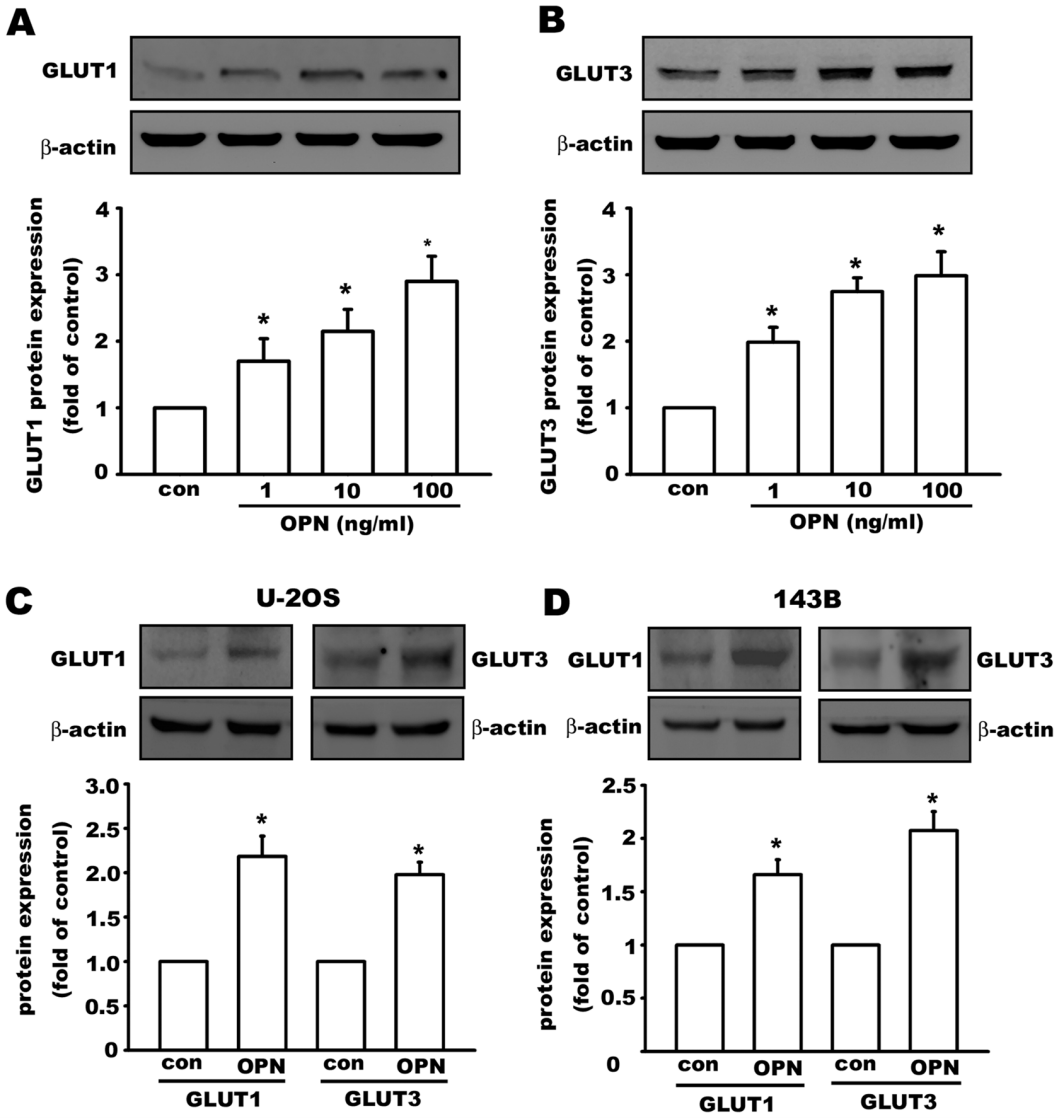
Osteopontin increases GLUT1 and GLUT3 expression in osteosarcoma cell lines. OPN (24 h) increased GLUT1 (A) and GLUT3 (B) protein levels in a concentration-dependent manner in MG63 osteosarcoma cells. OPN (10 ng/ml, 24 h) also increased GLUT1 and GLUT3 protein expression in U-2OS (C) and 143B (D) osteosarcoma cells. Data are presented as the mean ± S.E.M. (n = 4), *p≤0.05, as compared with the control group (con).

Because osteopontin is one of the hypoxia-inducible genes and a cancer progression marker, we performed further investigations of the effect of OPN on the regulation of glucose transporters. We observed that treatment with OPN for 24 h increased GLUT1 (Figure 3A) and GLUT3 (Figure 3B) protein expression in a concentration-dependent manner in MG63 osteosarcoma cells. GLUT1 and GLUT3 are also upregulated by OPN (10 ng/ml) treatment in two other osteosarcoma cell lines, U-2OS (Figure 3C) and 143B (Figure 3D). These results demonstrate that OPN can regulate glucose transporter expression in osteosarcoma.

### Increase of glucose uptake by OPN in osteosarcoma cells

**Figure 4:**
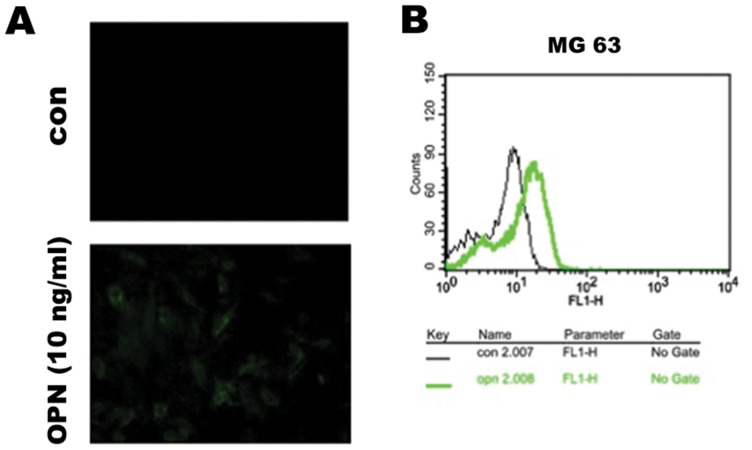
Osteopontin increases glucose uptake in MG63 osteosarcoma cells. 2-NBDG, a fluorescent d-glucose analog, was used as an indicator of glucose uptake. Note that treatment with OPN (100 ng/ml) for 24 h enhanced 2-NBDG uptake into MG63 cells, as shown by confocal microscopy (A) and flow cytometric analysis (B).

Because of the fact that OPN can upregulate the expression of glucose transporters in osteosarcoma cells, we further examined the effect of OPN on glucose uptake in MG63 cells. 2-NBDG, a fluorescent D-glucose analog for direct measurement of glucose uptake, was used to examine the effect of OPN on glucose uptake. Immunofluorescence showed that 2-NBDG uptake was increased following treatment with OPN (100 ng/ml, 24 h) (Figure 4A). Flow cytometric analysis showed that the fluorescence of 2-NBDG was right-shifted by treatment with OPN (100 ng/ml, 24 h) (Figure 4B). These results indicate that exogenous OPN can further increase glucose uptake into hypoxic osteosarcoma cells.

### Knockdown of osteopontin decreases the expression of glucose transporters and cell viability in osteosarcoma cells

**Figure 5:**
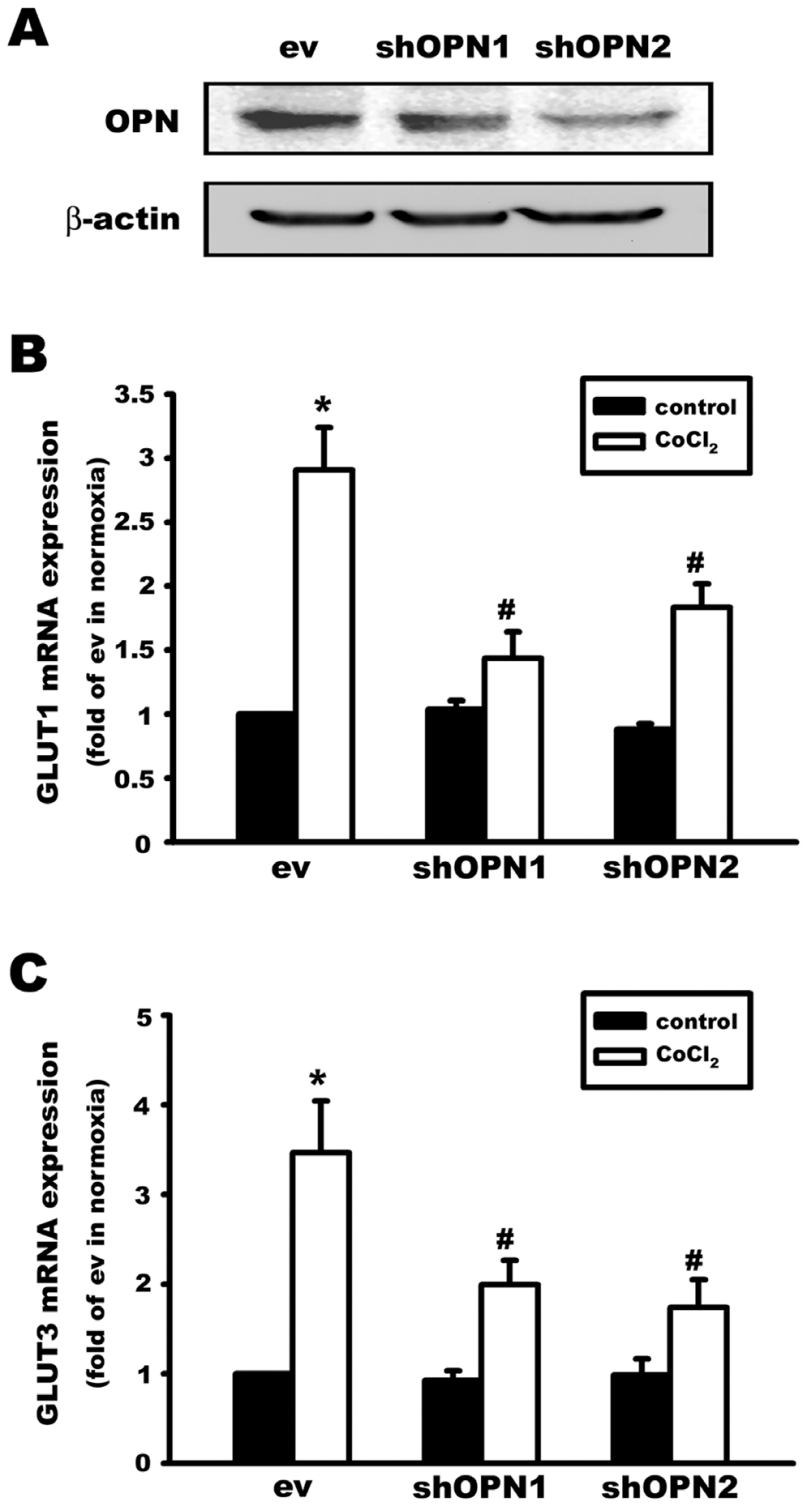
Knockdown of osteopontin decreases glucose transporters expression in a hypoxic osteosarcoma cell line. (A) Two OPN-shRNA plasmids (shOPN1 and shOPN2) and one empty vector (ev) plasmid were transiently transfected (24 h) in MG63 cells. OPN protein expression was downregulated by both shOPN1 and shOPN2. After treatment with the chemical hypoxia agent CoCl_2_ (100 µM, 6 h), GLUT1 (B) and GLUT3 (C) mRNA expression was markedly upregulated in the empty vector (ev) group. This effect was significantly antagonized by OPN knockdown (shOPN1 and shOPN2) in MG63 cells. Data are presented as the mean ± S.E.M. (n = 4), *p≤0.05, compared with the empty vector group (ev) in the control group, #p≤0.05, compared with the empty vector group (ev) in the CoCl_2_ treatment group.

The role of endogenously released OPN was investigated by OPN knockdown in osteosarcoma cells using shRNA transfection. Transfection with two sequences of OPN-specific shRNA (sh1 and sh2) for 24 h downregulated OPN protein expression compared with empty vector (ev) (Figure 5A). CoCl_2_ treatment (100 µM, 6 h) upregulated GLUT1 (Figure 5B) and GLUT3 (Figure 5C) mRNA expression in MG63 cells, which was antagonized by OPN shRNA transfection, indicating that endogenously released OPN is involved in hypoxia-induced GLUT1 and GLUT3 expression.

**Figure 6:**
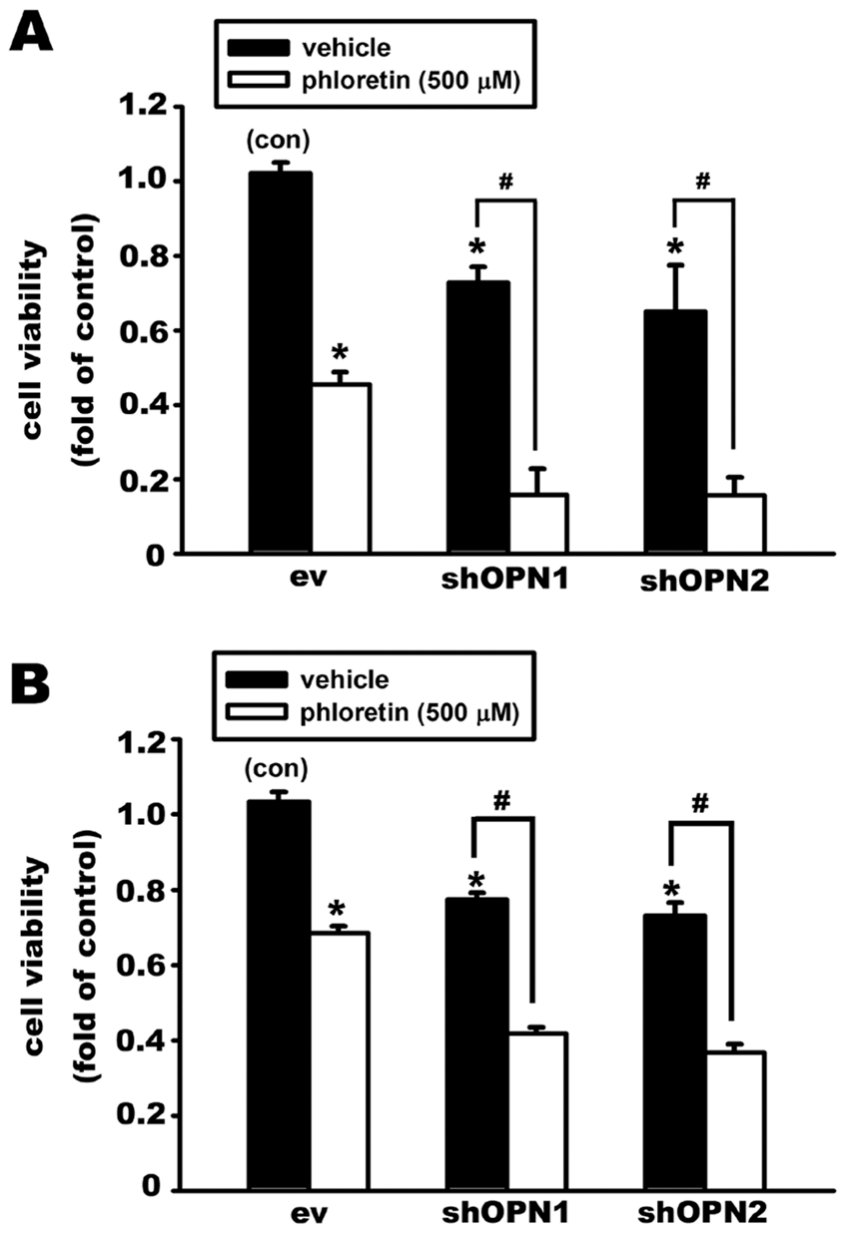
Knockdown of osteopontin enhances glucose transporter inhibitor phloretin-induced cell death in osteosarcoma cell lines. Knockdown of OPN expression by transient transfection of OPN-shRNA (shOPN1 and shOPN2) induced approximately 20% cell death in MG63 (A) and U-2OS (B) cells. Inhibition of glucose transporter activity by phloretin (500 µM, 24 h) also caused cell death in the empty vector (ev) group. The cytotoxic effect of phloretin was enhanced by OPN knockdown in both MG63 (A) and U-2OS (B) cells. Cell viability was measured by MTT assay. Data are presented as the mean ± S.E.M. (n = 4), *p≤0.05, compared with the control group (con), #p≤0.05, compared with respective vehicle-treated group.

In addition, OPN knockdown for 48 h decreased cell viability in both MG63 (Figure 6A) and U-2OS (Figure 6B) osteosarcoma cells. Treatment with phloretin (500 µM), a glucose transporter inhibitor, for 24 h also decreased cell viability in MG63 (Figure 6A) and U-2OS (Figure 6B) osteosarcoma cells. The apoptotic effect of phloretin was further enhanced in OPN knockdown cells. These results indicate that endogenously released OPN plays an important role in regulating GLUTs expression and cell survival in osteosarcoma cells.

### The αvβ3 integrin and MAPK pathways are involved in osteopontin-induced glucose transporter upregulation in osteosarcoma cells

**Figure 7:**
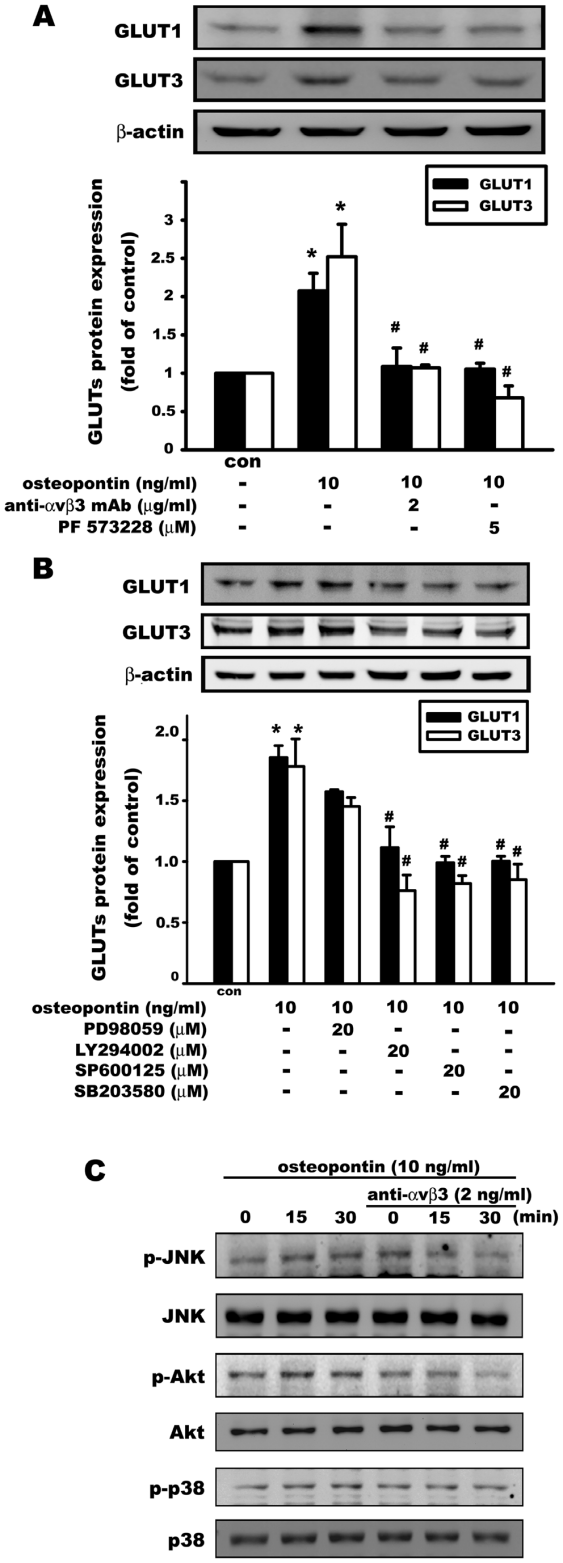
Osteopontin regulates GLUT1 and GLUT3 expression via the αvβ3 integrin and MAPK pathways in osteosarcoma cells. OPN (10 ng/ml) increased GLUT1 and GLUT3 protein expression in MG63 cells. This effect was significantly antagonized by pretreatment with an anti-αvβ3 mAb (2 µg/ml) and PF573228 (5 µM, FAK inhibitor) (A). (B) MG63 cells were pretreated with PD98059 (20 µM), LY294002 (20 µM), SP600125 (20 µM), and SB203580 (20 µM) for 30 min and then stimulated with OPN (10 ng/ml, 24 h). OPN-induced increase of GLUT1 and GLUT3 protein expression was significantly antagonized by LY294002, SP600125, and SB203580. (C) OPN (10 ng/ml) increased the phosphorylation of AKT, JNK, and p38 in a time-dependent manner, and pretreatment with an anti-αvβ3 mAb (2 µg/ml) inhibited OPN-induced AKT, JNK, and p38 phosphorylation. Data are presented as the mean ± S.E.M. (n = 3). *p≤0.05, compared with the control group (con), #p≤0.05, compared with OPN treatment alone.

Osteopontin, a secreted adhesive glycoprotein with a functional RGD cell-binding domain, interacts primarily with the αvβ3 integrin. As shown in Figure 7A, the OPN-induced increase of GLUT1 and GLUT3 protein expression was significantly antagonized by a αvβ3 monoclonal antibody (2 µg/ml) and PF573228 (focal adhesion kinase (FAK) inhibitor, 5 µM) in MG63 cells, indicating that OPN increased GLUT1 and GLUT3 expression via αvβ3 integrin and caused the activation of the downstream protein kinase FAK. OPN-induced increase of GLUT1 and GLUT3 protein expression was also markedly inhibited by the PI3K inhibitor LY294002 (20 µM), the JNK inhibitor SP600125 (20 µM), and the p38 inhibitor SB203580 (20 µM), whereas the ERK inhibitor PD98059 (20 µM) did not affect the OPN-induced expression of GLUT1 and GLUT3 (Figure 7B). It was also found that OPN time-dependently phosphorylated phosphoinositide-3 kinase (PI3K/AKT), Jun-amino-terminal kinase (JNK), and the p38 pathway, which were antagonized by an αvβ3 integrin monoclonal antibody (Figure 7C). These results indicate that several kinases, including PI3K/AKT, JNK, and p38, are involved in the regulation of glucose transporters by OPN via αvβ3 integrin.

### The synergistic cytotoxic effect of chemotherapy drugs in combination with a GLUT inhibitor in osteosarcoma cells

**Figure 8:**
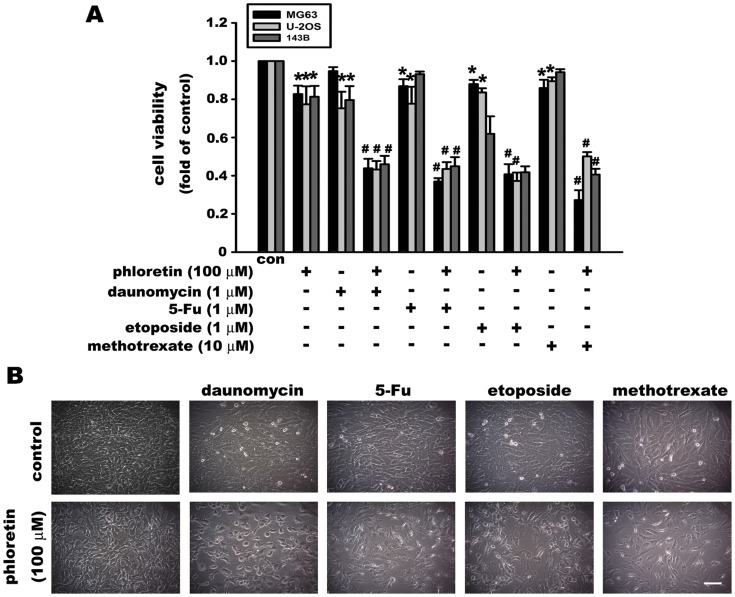
The cytotoxic effect of chemotherapeutic drugs is enhanced by combination with a glucose transporter inhibitor. (A) Treatment of MG63 cells with phloretin (100 µM), daunomycin (1 µM), 5-Fu (10 µM), etoposide 10 µM, or methotrexate (10 µM) alone for 24 h induced a low level of cell death. However, the combination of phloretin with chemotherapeutic drugs (daunomycin, 5-Fu, etoposide, and methotrexate) markedly increased cell death in three osteosarcoma cell lines: MG63, U-2OS, and 143B. Representative photographs are shown in panel B. Data are presented as the mean ± S.E.M. (n = 4). *p≤0.05, compared with the control (con), #p≤0.05, compared with the respective treatment of the chemotherapeutic drug alone.

Phloretin is a competitive inhibitor of glucose transporters that has potent antioxidant activity, as well as anti-proliferative and apoptotic effects in cancer cells, such as hepatocellular carcinoma (HepG2) [28], colon cancer (HT-29) [29], melanoma (B16) [30], and breast cancer (MCF10A). Here we used a low concentration of phloretin (100 µM) in combination with chemotherapeutic drugs, such as daunomyacin (1 µM), 5-Fu (10 µM), etoposide (10 µM), and methotrexate (10 µM), in the osteosarcoma cell lines MG63, U-2OS, and 143B. Treatment with phloretin (100 µM), daunomyacin (1 µM), 5-Fu (10 µM), etoposide (10 µM), or methotrexate (10 µM) alone for 24 h caused only 20% cell death. However, the combination of phloretin with these chemotherapeutic drugs markedly increased cell death (>50%) in all three osteosarcoma cell lines (Figure 8A). Representative images of phloretin in combination with chemotherapeutic drugs are shown in Figure 8B. These results indicate that the addition of a low dose of glucose transporter inhibitor with chemotherapeutic drugs can enhance cytotoxicity in osteosarcoma cells.

## Discussion

In this study we demonstrate that both osteopontin and glucose transporters are crucial factors in osteosarcoma. Endogenously released OPN regulated GLUTs expression in hypoxia, which was reversed by knockdown of OPN. Inhibition of the function of OPN and GLUTs induced cell death in osteosarcoma cell lines. Combination of a GLUT inhibitor and chemotherapy drugs exerted a synergistic apoptotic effect in osteosarcoma.

Cobalt chloride (CoCl_2_), a hypoxia-mimetic agent, has been demonstrated to inhibit the prolyl hydroxylase domain-containing enzymes (PHDs) [31], which plays a key role in oxygen-dependent degradation of the transcription factor, to activate hypoxia-mediated signaling by stabilizing hypoxia-inducible transcription factor-1α (HIF-1α) [32]–[34]. Here we used CoCl_2_ to mimic the hypoxic condition, an inevitable cellular stress experienced during tumor progression and solid tumor development [35], [36]. During hypoxia, HIF-1α degradation is inhibited and its activity is increased. Increase of HIF-1α activity is mediated through the PI3K-Akt and MAPK signaling pathways [37], [38]. OPN is a bone-associated extracellular matrix protein that is produced by numerous cell types, such as osteoblasts, osteoclasts, T lymphocytes, NK cells, and epithelial cells. OPN influences normal physiological processes, including bone resorption, wound healing, tissue remodeling, and vascularization [39]. OPN has also been shown to be involved in all stages of cancer progression; for instance, tumor invasion, angiogenesis, and metastasis [40]–[42]. Here, we found that CoCl_2_-induced hypoxia upregulated OPN mRNA and protein expression in osteosarcoma cells. The overexpression of GLUTs is requisite for cell proliferation, like that seen in cancer, in order to increase the energy supply. We also demonstrated that GLUTs can be upregulated by both CoCl_2_ and OPN.

Because GLUTs were upregulated by OPN, glucose uptake was evaluated using a fluorescent d-glucose analog, 2-NBDG, in MG63 osteosarcoma cells with confocal microscopy and flow cytometry [43], [44]. The intracellular fluorescence intensity of 2-NBDG was enhanced by treatment with OPN in MG63, indicating that OPN increases nutrient availability to osteosarcoma cells. This effect was mediated by αvβ3 integrin, FAK phosphorylation, and the AKT, JNK, and p38 MAPK pathways.

Knockdown of OPN expression was performed in the osteosarcoma cell lines MG63 and U-2OS using two different shRNA plasmids. Cell survival was decreased by approximately 20% in OPN knockdown cells. Hypoxia-induced expression of GLUTs was also inhibited by OPN knockdown. These results indicate that endogenously released OPN can regulate GLUTs expression, glucose uptake, and the survival of osteosarcoma cells.

Phloretin, a natural product, exists in the fruit trees in glucosidic form, namely phloridzin. Both of phloretin and phloridizin are present in apple and pulp [45]. Phloretin amounts to 12.5 µg/mL in apple juice and 219 µg/mL in carrot juice. Phloretin has a marked effect on the survival of colon cancer cells at concentrations as low as 50 µmol/L [46]. It has been demonstrated that phloretin, isoliquiritigenin and other hydroxylated chalcones had cytotoxic activity by inducing collapse of mitochondrial membrane potential and increasing oxygen uptake [47]. Phloretin (the aglucon of phlorizin) is reported to induce human liver cancer cell apoptosis and exert significant anti-tumor effects in HepG2 xenograft animal model by administering phloretin (10 mg/kg) intraperitoneally [28]. Although phlorizin makes the tumor cells impermeable to glucose, it also makes all other cells all over the body impermeable to glucose cells. However, phlorizin derivatives can sensitize the cancer cells for treatment with heat and other modalities in patients [48]. Here we used phloretin in osteosarcoma. Inhibition of GLUTs by 500 µM phloretin induced approximately 50% cell death in osteosarcoma cell lines. OPN knockdown enhanced the cell death induced by phloretin to 80%, indicating that phloretin-induced cell death was more sensitive in OPN knockdown osteosarcoma cells. This apoptosis enhancing effect also appeared at low dose of phloretin (100 µM, 6 h) in both MG63 (Figure S1A) and U-2OS (Figure S1B) osteosarcoma cells, indicating that OPN knockdown sensitized the tumor cells to phloretin-induced apoptosis. Both glucose and OPN are important to osteosarcoma cell survival. Glucose uptake is related to cancer cell survival and drug sensitivity. For instance, the decrease in glucose uptake at an early time point after a high dose of cisplatin reflects cisplatin chemosensitivity in ovarian cancer cells [49]. Phloretin also sensitizes cancer cells to daunorubicin and overcomes drug resistance in hypoxia [50]. Chemotherapy is used in the treatment of osteosarcoma. As shown above, osteosarcoma is sensitive to the glucose transporter inhibitor phloretin. Moreover, combination of a low dose of glucose transporter inhibitor with chemotherapy drugs markedly enhanced cell death in osteosarcoma cell lines. These results indicate that a combination therapy of a low dose of a GLUTs inhibitor and a cytotoxic drug may offer a new therapeutic option to osteosarcoma patients.

In conclusion, endogenously released OPN is important for the regulation of GLUT1 and GLUT3 expression in osteosarcoma. Inhibition of glucose uptake by a transporter inhibitor can induce cell death in osteosarcoma. Furthermore, the combination of a low dose of phloretin with chemotherapeutic drugs markedly enhanced cell death in osteosarcoma cells.

## Supporting Information

Figure S1 **Low dose of phloretin enhances cell death of osteosarcoma in osteopontin knockdown cells.** Knockdown of OPN expression (24 h) by transient transfection of OPN-shRNA (shOPN1 and shOPN2) induced approximately 10% cell death in MG63 (A) and U-2OS (B) cells. Inhibition of glucose transporter activity by phloretin (100 µM, 6 h) caused cell death in the empty vector (ev) group. The cytotoxic effect of phloretin was enhanced by OPN knockdown in both MG63 (A) and U-2OS (B) cells after short duration of 6 h treatment. Cell viability was measured by MTT assay. Data are presented as the mean ± S.E.M. (n = 4). *p≤0.05, compared with control group (con); #p≤0.05, compared with respective vehicle-treated group.
(DOCX)
