# Supplementary material for: Osteopontin Upregulates the Expression of Glucose Transporters in Osteosarcoma Cells
Source: PLoS One. 2014 Oct 13;9(10):e109550. doi: 10.1371/journal.pone.0109550 (PMC4195676; doi:10.1371/journal.pone.0109550)
Supplement: Figure S1 — Low dose of phloretin enhances cell death of osteosarcoma in osteopontin knockdown cells. Knockdown of OPN expression (24 h) by transient transfection of OPN-shRNA (shOPN1 and shOPN2) induced approximately 10% cell death in MG63 (A) and U-2OS (B) cells. Inhibition of glucose transporter activity by phloretin (100 µM, 6 h) caused cell death in the empty vector (ev) group. The cytotoxic effect of phloretin was enhanced by OPN knockdown in both MG63 (A) and U-2OS (B) cells after short duration of 6 h treatment. Cell viability was measured by MTT assay. Data are presented as the mean ± S.E.M. (n = 4). *p≤0.05, compared with control group (con); #p≤0.05, compared with respective vehicle-treated group. (DOCX) [file pone.0109550.s001.docx]

**SUPPORTING INFORMATION S1**

**Figure S1**


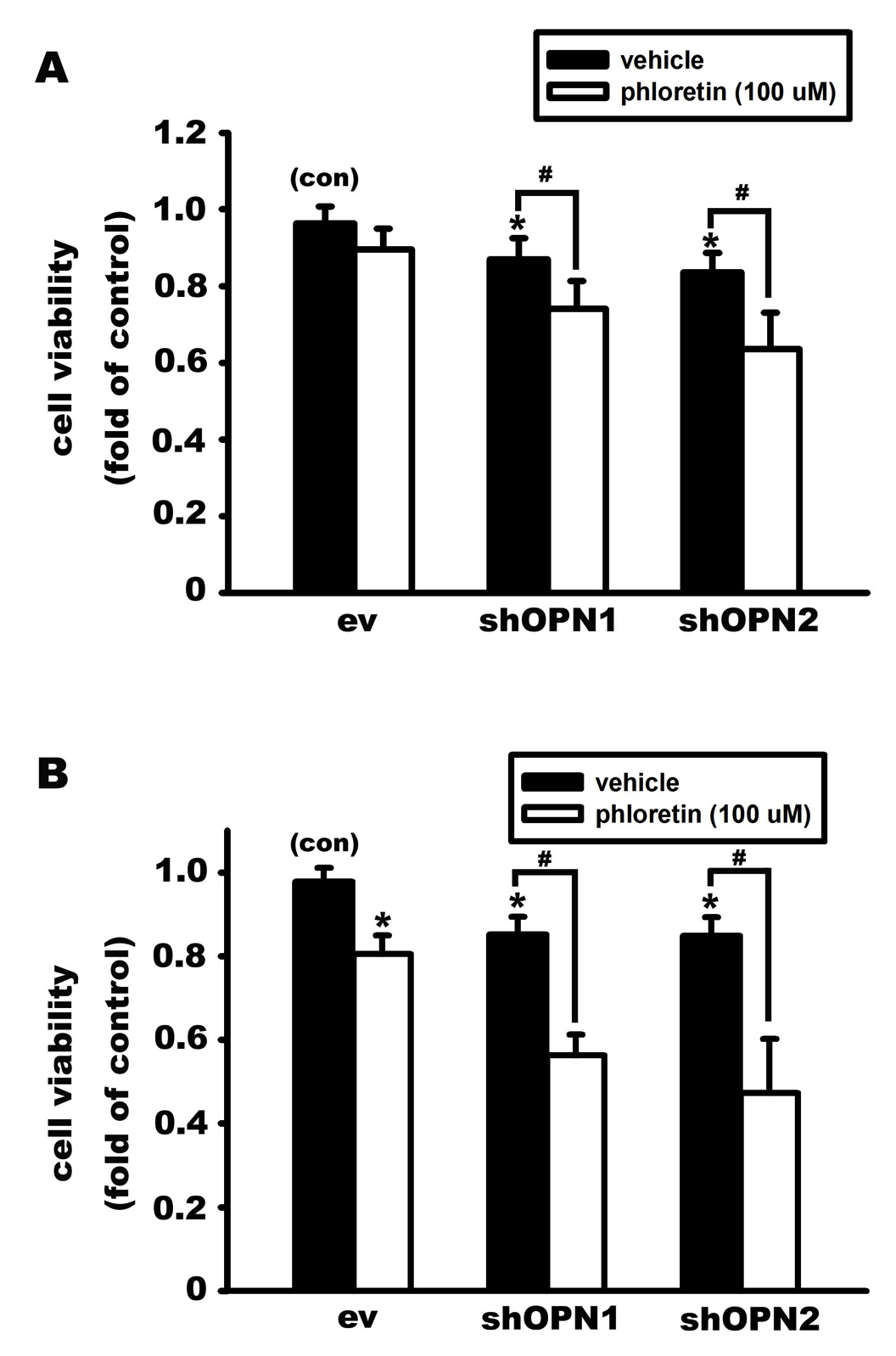


**Supporting Information Legend**

*Figure S1. Low dose of phloretin enhances cell death of osteosarcoma in osteopontin knockdown cells.*

Knockdown of OPN expression (24h) by transient transfection of OPN-shRNA (shOPN1 and shOPN2) induced approximately 10% cell death in MG63 (A) and U-2OS (B) cells. Inhibition of glucose transporter activity by phloretin (100 μM, 6 h) caused cell death in the empty vector (ev) group. The cytotoxic effect of phloretin was enhanced by OPN knockdown in both MG63 (A) and U-2OS (B) cells after short duration of 6h treatment. Cell viability was measured by MTT assay. Data are presented as the mean ± S.E.M. (n = 4). *p ≤ 0.05, compared with control group (con) ; #p ≤ 0.05, compared with respective vehicle-treated group.
